# Deep learning improves test–retest reproducibility of regional strain in echocardiography

**DOI:** 10.1093/ehjimp/qyae092

**Published:** 2024-10-23

**Authors:** John Nyberg, Andreas Østvik, Ivar M Salte, Sindre Olaisen, Sigve Karlsen, Thomas Dahlslett, Erik Smistad, Torfinn Eriksen-Volnes, Harald Brunvand, Thor Edvardsen, Kristina H Haugaa, Lasse Lovstakken, Havard Dalen, Bjørnar Grenne

**Affiliations:** Department of Circulation and Medical Imaging, NTNU–Norwegian University of Science and Technology, Box 8905, 7491 Trondheim, Norway; ProCardio Center for Innovation, Department of Cardiology, Oslo University Hospital, Rikshospitalet, Box 4950, 0424 Oslo, Norway; Department of Circulation and Medical Imaging, NTNU–Norwegian University of Science and Technology, Box 8905, 7491 Trondheim, Norway; Medical Image Analysis, Health Research, SINTEF Digital, Trondheim, Norway; ProCardio Center for Innovation, Department of Cardiology, Oslo University Hospital, Rikshospitalet, Box 4950, 0424 Oslo, Norway; Department of Radiology, Akershus University Hospital, Lørenskog, Norway; Department of Circulation and Medical Imaging, NTNU–Norwegian University of Science and Technology, Box 8905, 7491 Trondheim, Norway; Department of Medicine, Hospital of Southern Norway, Arendal, Norway; Department of Medicine, Hospital of Southern Norway, Arendal, Norway; Department of Circulation and Medical Imaging, NTNU–Norwegian University of Science and Technology, Box 8905, 7491 Trondheim, Norway; Medical Image Analysis, Health Research, SINTEF Digital, Trondheim, Norway; Department of Circulation and Medical Imaging, NTNU–Norwegian University of Science and Technology, Box 8905, 7491 Trondheim, Norway; Clinic of Cardiology, St. Olavs Hospital, Trondheim, Norway; Department of Medicine, Hospital of Southern Norway, Arendal, Norway; ProCardio Center for Innovation, Department of Cardiology, Oslo University Hospital, Rikshospitalet, Box 4950, 0424 Oslo, Norway; Faculty of Medicine, University of Oslo, Oslo, Norway; ProCardio Center for Innovation, Department of Cardiology, Oslo University Hospital, Rikshospitalet, Box 4950, 0424 Oslo, Norway; Faculty of Medicine, University of Oslo, Oslo, Norway; Department of Circulation and Medical Imaging, NTNU–Norwegian University of Science and Technology, Box 8905, 7491 Trondheim, Norway; Department of Circulation and Medical Imaging, NTNU–Norwegian University of Science and Technology, Box 8905, 7491 Trondheim, Norway; Clinic of Cardiology, St. Olavs Hospital, Trondheim, Norway; Department of Medicine, Levanger Hospital, Nord-Trøndelag Hospital Trust, Levanger, Norway; Department of Circulation and Medical Imaging, NTNU–Norwegian University of Science and Technology, Box 8905, 7491 Trondheim, Norway; Clinic of Cardiology, St. Olavs Hospital, Trondheim, Norway

**Keywords:** artificial intelligence, consistency, precision, myocardial deformation

## Abstract

**Aims:**

The clinical utility of regional strain measurements in echocardiography is challenged by suboptimal reproducibility. In this study, we aimed to evaluate the test–retest reproducibility of regional longitudinal strain (RLS) per coronary artery perfusion territory (RLS_Territory_) and basal-to-apical level of the left ventricle (RLS_Level_), measured by a novel fully automated deep learning (DL) method based on point tracking.

**Methods and results:**

We measured strain in a dual-centre test–retest data set that included 40 controls and 40 patients with suspected non-ST elevation acute coronary syndrome. Two consecutive echocardiograms per subject were recorded by different operators. The reproducibility of RLS_Territory_ and RLS_Level_ measured by the DL method and by three experienced observers using semi-automatic software (2D Strain, EchoPAC, GE HealthCare) was evaluated as minimal detectable change (MDC). The DL method had MDC for RLS_Territory_ and RLS_Level_ ranging from 3.6 to 4.3%, corresponding to a 33–35% improved reproducibility compared with the inter- and intraobserver scenarios (MDC 5.5–6.4% and 4.9–5.4%). Furthermore, the DL method had a lower variance of test–retest differences for both RLS_Territory_ and RLS_Level_ compared with inter- and intraobserver scenarios (all *P* < 0.001). Bland–Altman analyses demonstrated superior reproducibility by the DL method for the whole range of strain values compared with the best observer scenarios. The feasibility of the DL method was 93% and measurement time was only 1 s per echocardiogram.

**Conclusion:**

The novel DL method provided fully automated measurements of RLS, with improved test–retest reproducibility compared with semi-automatic measurements by experienced observers. RLS measured by the DL method has the potential to advance patient care through a more detailed, more efficient, and less user-dependent clinical assessment of myocardial function.

## Graphical Abstract

**Graphical Abstract:**
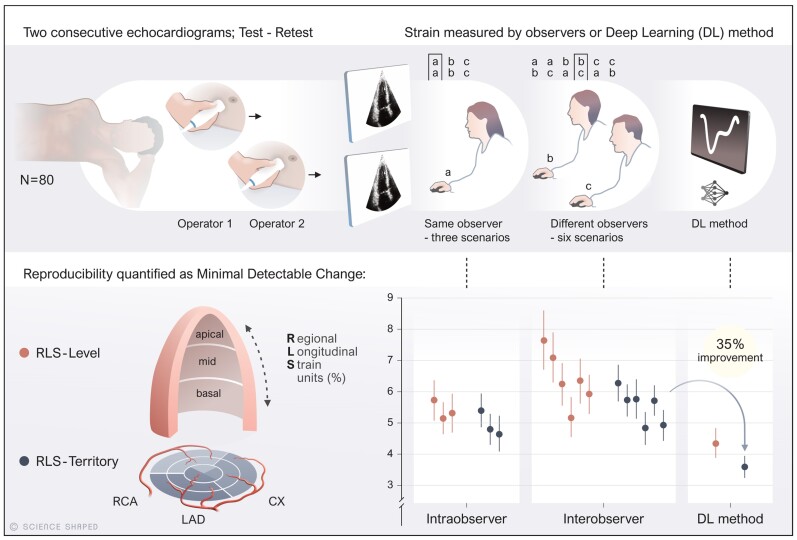
Deep learning improves reproducibility of regional strain in echocardiography. An overview of included subjects, echocardiographic recordings, strain measurements, and main results. The error bars represent 95% confidence intervals for estimates of minimal detectable change. RCA, right coronary artery; LAD, left anterior descending coronary artery; CX, circumflex coronary artery.

## Introduction

Echocardiographic measurement of left ventricular (LV) global longitudinal strain (GLS) is increasingly utilized as a supplement to ejection fraction (EF) for the quantification of global LV function. The incremental prognostic and diagnostic information of GLS has made it a guideline-recommended part of routine echocardiographic examinations.^1–3^ However, GLS is a regularized global mean, and valuable information about regional deformation is lost. Although existing semi-automatic softwares also report regional longitudinal strain (RLS), these measurements have high variability,^4–8^ and are therefore not recommended by current guidelines.^1^ To reduce measurement variability and time consumption of strain measurements, we previously developed an artificial intelligence method based on deep learning (DL) for fully automated measurements of GLS.^9,10^ We have now advanced this method by incorporating a novel learning-based point tracking DL technology instead of optical flow–based motion estimation. We hypothesize that this improves the estimation of regional myocardial deformation and thereby increases the precision of RLS measurements. Therefore, in this study, we aim to evaluate test–retest reproducibility of RLS per coronary artery perfusion territory (RLS_Territory_) and the basal-to-apical level of the LV (RLS_Level_) by the DL method using semi-automatic measurements conducted by experienced observers as reference.

## Methods

### Study design

We used a comprehensive dual-centre test–retest data set consisting of 40 subjects included in the population-based Nord-Trøndelag Health Study—Echocardiographic sub-study (HUNT4Echo) during 2018, and 40 subjects included in the clinical echocardiographic study of patients with previously suspected non-ST elevation acute coronary syndrome from the Hospital of Southern Norway, Arendal, during 2013 (further details are provided in Supplementary data online, *Methods*).^11,12^ These two studies were approved by the Regional Committees for Medical and Health Research Ethics in Central and South-Eastern Norway, respectively. The reproducibility of GLS, but not RLS, measured by a previous generation of the DL method was evaluated in this data set.^13^ Two echocardiographic recordings per subject were recorded consecutively to minimize physiological variability between recordings. Thus, the total data set constituted 160 echocardiograms.

### Echocardiographic acquisitions

The echocardiograms were acquired according to recommendations by the European Association of Cardiovascular Imaging and the American Society of Echocardiography.^1^ Subjects were positioned in the left lateral decubital position and examined using Vivid E95 (HUNT4Echo) and Vivid 7 (Hospital of Southern Norway) scanners (GE HealthCare, Horten, Norway). One experienced sonographer (K.S.) and one experienced cardiologist (T.E.-V.), with >2000 recordings and readings each, acquired the echocardiograms at HUNT4Echo, whereas one cardiologist (T.D.), with >300 recordings and >750 readings, and one cardiology resident (S.K.), with >150 recordings and >300 readings, acquired the echocardiograms at the Hospital of Southern Norway. At each centre, all subjects were scanned once by each operator, and the echocardiograms consisted of at least three cardiac cycles per recorded view. The LV-focused apical four-chamber (4CH), two-chamber (2CH), and long-axis (APLAX) views were used for strain measurements. The frame rate was 77 ± 9 frames/s.

### Strain measurements by the semi-automatic method

Three experienced observers (J.N., I.M.S., and S.O.) measured longitudinal strain in the whole data set using the semi-automatic 2D Strain speckle tracking application (EchoPAC SWO version 204, GE HealthCare, Horten, Norway). Each observer measured strain in one cardiac cycle per view where the region of interest (ROI) was optimized to cover the LV myocardium, while avoiding inclusion of the papillary muscles, trabeculations, and pericardium. Appropriate tracking at the segmental level was visually confirmed by ensuring that the ROI consistently followed the movement of the underlying myocardium. End systole was defined as the frame of aortic valve closure in the APLAX view, and the same timing was used in the 4CH and 2CH views. End diastole was defined as the application default, but changed to the frame of mitral valve closure in the APLAX view if needed.

### Strain measurements by the DL method

The automated DL method measured longitudinal strain in the three apical views without any observer input. Briefly, the DL method constitutes a fully automated pipeline for myocardial strain measurements constructed from four DL models. The models perform cardiac view classification, event detection, segmentation, and tracking.^9,10,13^ Technical details of the DL method have been previously described in detail.^9^ Compared with previous versions, the current method incorporates a novel learning-based point tracking algorithm instead of the dense optical flow–based motion estimator.^14^ Building upon our previous studies, this approach was first trained on simulated data consisting of synthetic non-echo images,^9^ along with 203 simulated ultrasound sequences of 4CH views.^15,16^ We fine-tuned the model on real echocardiographic videos obtained from an independent data set of 379 subjects, consisting of the three apical views.

Furthermore, the DL pipeline was enhanced with an additional component to automatically evaluate tracking and image quality. The tracking quality of reference data was annotated and quality-assured by two experts who scored image and tracking quality per segment. Leveraging features extracted from the novel point tracking network as input and the image and tracking quality annotations as ground truth, we trained a linear layer with sigmoid activation, employing cross-entropy loss as the learning criterion. This new component ensured that only strain measurements from segments with at least acceptable image and tracking quality were included. The DL method measured strain in all available recorded cycles.

### Calculation of RLS and GLS

RLS was assessed as RLS_Territory_, corresponding to the theoretical perfusion areas of the three main epicardial coronary arteries, and as RLS_Level_, corresponding to the base, mid-, and apical levels of the LV. The LV apex constitutes a small myocardial mass with highly variable coronary artery perfusion, and to reduce its influence on strain values we used the guideline-recommended 16-segment LV model with four apical segments (see Supplementary data online, *Figure S1*).^1^ This was achieved by averaging the lateral and posterior, and septal and anteroseptal apical segments, respectively.^17^ For calculation of RLS_Territory_, we averaged segments belonging to the perfusion territories of the left anterior descending-, circumflex- and right coronary artery, respectively (see Supplementary data online, *Figure S1*).^17^ Similarly, for calculation of RLS_Level_, we averaged the basal-, mid-, and apical myocardial segments. For GLS, the 16 segmental strain values were averaged. Calculations of RLS and GLS were similar for the semi-automatic and DL methods. Segments with unacceptable tracking quality were discarded and not included in the calculation of strain values. All strain values are reported as absolute numbers of peak systolic strain.

### Statistics

Continuous normally distributed data are presented as mean ± standard deviation (SD) and categorical data as numbers (%). Data distributions were assessed by visual inspections of histograms. Strain measurements in the two consecutive echocardiograms were compared for the DL method, between different observers (interobserver) and between the same observer (intraobserver) to define the test–retest variability of each measurement scenario.

The test–retest variability is referred to as reproducibility and was quantified using:

1. Minimal detectable change (MDC), calculated as 1.96 × √ 2 times the standard error of measurement (SEM). The SEM was calculated as the square root of average within-subject variances.^18^
2. Intraclass correlation coefficient (ICC 2,1).^19^
3. Bland–Altman analyses, reported as bias and 95% limits of agreements (LOAs).^20^
4. Within-subject coefficient of variation (CoV), calculated as the SD divided by the mean.
5. Variances of within-subject differences, compared between methods using *F*-tests.

95% confidence intervals (CIs) for MDC were generated by bootstrapping with 1000 repetitions. The feasibility was calculated as the ratio of echocardiograms with successful strain measurements. Strain values measured by the DL method and the three observers were compared by analysis of variance, with *P* values adjusted using the Tukey correction. Data were analysed using R software version 4.2.2 (packages: ggplot2 and gtsummary) and Python version 3.8.10 (packages: pandas, pingouin, and numpy). A two-sided *P* value <0.05 was considered statistically significant.

## Results

### Clinical characteristics

The study population consisted of 80 subjects aged 61 ± 12 years (range 28–88 years) with LVEF 55 ± 9% (range 29–72%). Thirty-two (40%) were females. Cardiovascular risk factors including smoking, hypertension, diabetes mellitus, and hypercholesterolaemia were common, and 34 (42%) had established coronary artery disease (*Table 1*).

**Table 1:** Clinical characteristics of the study population

Variables	Study population *N* = 80
Male sex	48 (60%)
Age, years	61 ± 12
Height, cm	173 ± 8
Weight, kg	82 ± 15
Body mass index, kg/m^2^	27.4 ± 4.0
Body surface area, m^2^	1.96 ± 0.19
Systolic blood pressure, mmHg	137 ± 19
Diastolic blood pressure, mmHg	78 ± 11
Mean arterial pressure, mmHg	99 ± 13
Heart rate, beats/min	65 ± 11
Current smoker	8 (10%)
Coronary artery disease	34 (43%)
Hypertension	43 (54%)
Diabetes mellitus	10 (13%)
Hypercholesterolaemia	45 (56%)
LVEF, %	55 ± 9
LVEDV, mL	119 ± 30

Data are presented as mean ± SD or *n* (%).

LVEF, left ventricular ejection fraction; LVEDV, left ventricular end-diastolic volume.

### Test–retest reproducibility of strain measurements

**Figure 1:**
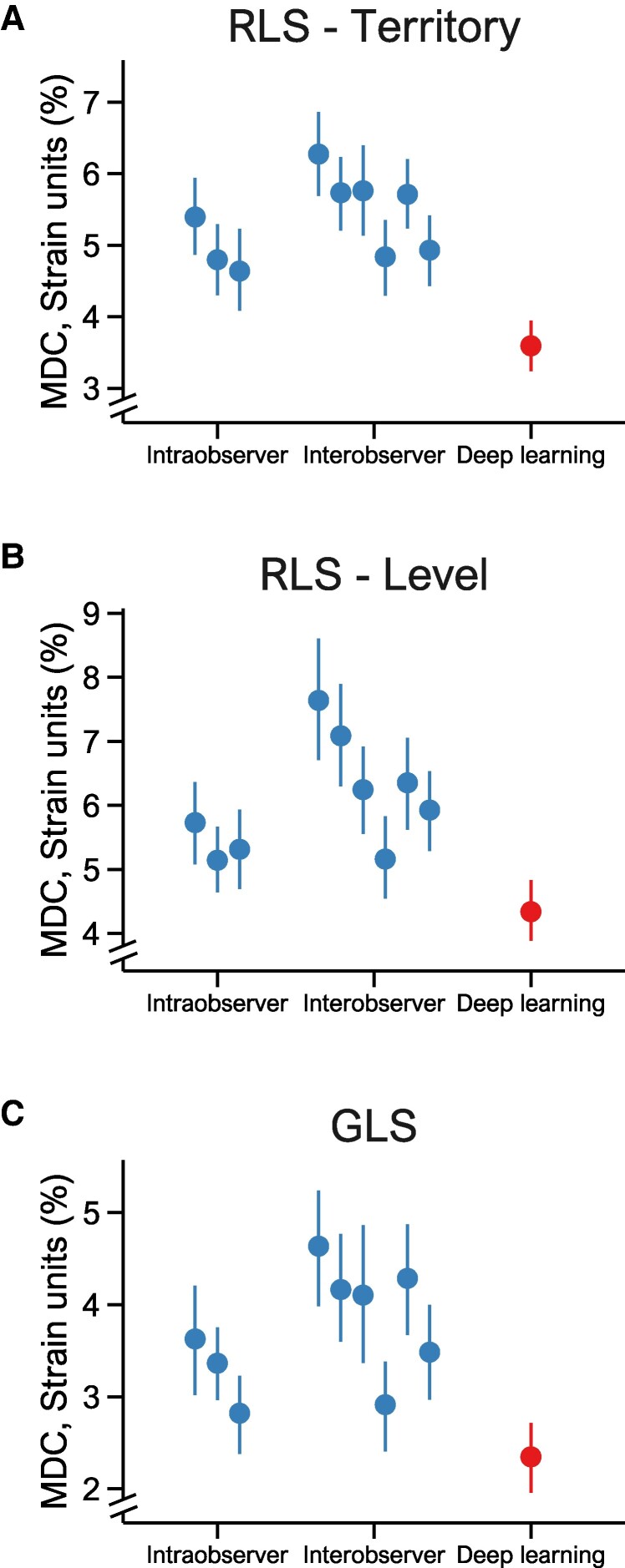
MDC for strains by regions, methods, and observers. MDC estimates and 95% CIs for RLS_Territory_ (*A*), RLS_Level_ (*B*), and GLS (*C*) in repeated recordings for the intraobserver scenarios, interobserver scenarios, and the DL method. RLS, regional longitudinal strain; GLS, global longitudinal strain.

The DL method demonstrated superior reproducibility across all RLS measurements compared with the human observers as assessed by MDC, ICC, bias, LOA, and CoV (*Table 2*, Supplementary data online, *Table S1*). For RLS_Territory_ by the DL method, the MDC was 3.6%, corresponding to a 35% improvement in reproducibility compared with the interobserver MDC of 5.5% (*Table 2*, *Graphical Abstract*, and *Figure 1*). Similarly, for RLS_Level_ by the DL method, the MDC was 4.3%, corresponding to a 33% improvement compared with the interobserver MDC of 6.4%. Furthermore, for GLS by the DL method, the MDC was 2.3%, corresponding to a 41% improvement compared with the interobserver MDC of 3.9%.

**Table 2:** Test–retest reproducibility metrics for strain measurements by regions, methods, and observers

	Intraobserver	Interobserver	Deep learning
Regional longitudinal strain Territory			
MDC	4.9 (4.6–5.4)	5.5 (4.8–6.3)	3.6
ICC	0.80 (0.81–0.89)	0.80 (0.71–0.85)	0.90
Bias	0.0 (−0.4 to 0.2)	0.0 (−1.4 to 1.4)	0.0
LOA (lower)	−5.0 (−5.3 to −4.5)	−5.2 (−7 to −3.6)	−3.6
LOA (higher)	4.9 (4.3–5.5)	5.1 (4.2–6.4)	3.6
CoV	6 (6–6)	7 (6–7)	5
Regional longitudinal strain Level			
MDC	5.4 (5.1–5.7)	6.4 (5.2–7.6)	4.3
ICC	0.80 (0.8–0.86)	0.70 (0.66–0.85)	0.90
Bias	−0.1 (−0.5 to 0.1)	−0.1 (−1.6 to 1.4)	0.0
LOA (lower)	−5.5 (−5.7 to −5.3)	−6.1 (−8.6 to −4.4)	−4.4
LOA (higher)	5.3 (4.6–5.8)	5.9 (5.2–7.1)	4.4
CoV	6 (6–6)	7 (6–8)	6
Global longitudinal strain			
MDC	3.3 (2.8–3.6)	3.9 (2.9–4.6)	2.3
ICC	0.80 (0.81–0.89)	0.80 (0.71–0.86)	0.90
Bias	−0.1 (−0.5 to 0.1)	0.0 (−1.4 to 1.4)	0.0
LOA (lower)	−3.3 (−3.7 to −2.7)	−3.5 (−5.1 to −1.9)	−2.4
LOA (higher)	3.2 (2.8–3.8)	3.4 (2.3–4.7)	2.4
CoV	4 (4–4)	5 (4–6)	3

The mean (range) of all metrics is provided for the observer scenarios. All metrics are reported as strain percentages (%), except ICC, which is unitless and CoV, which is a relative percentage.

MDC, minimal detectable change; ICC, intraclass correlation coefficient; LOA, limits of agreement; CoV, coefficient of variation.

**Figure 2:**
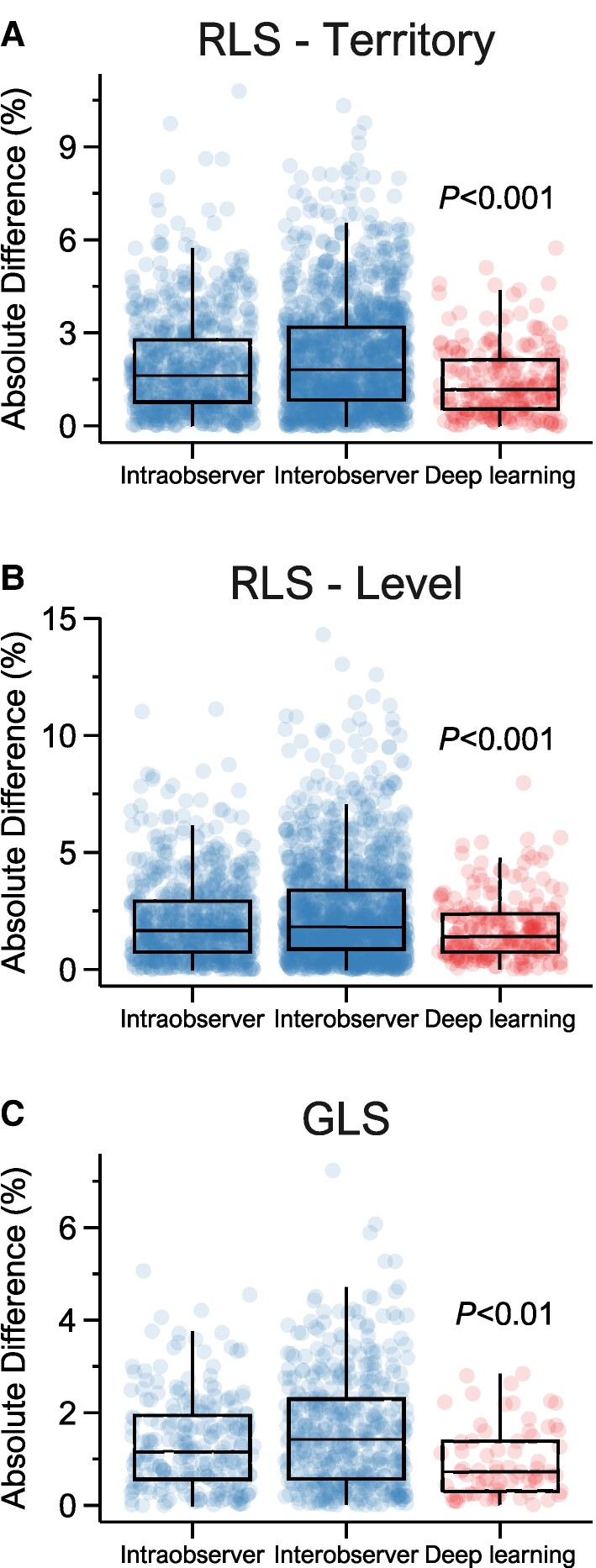
Absolute differences in strain values in repeated recordings by regions, methods, and observers. Boxplots with overlaying data points of RLS_Territory_ (*A*), RLS_Level_ (*B*), and GLS (*C*) measurements in repeated recordings for the intraobserver and interobserver scenarios using the semi-automatic method and the DL method. The *P* values for *F*-tests of variances of differences between DL and observer scenarios are shown. Abbreviations: as in *Figure 1*.

**Figure 3:**
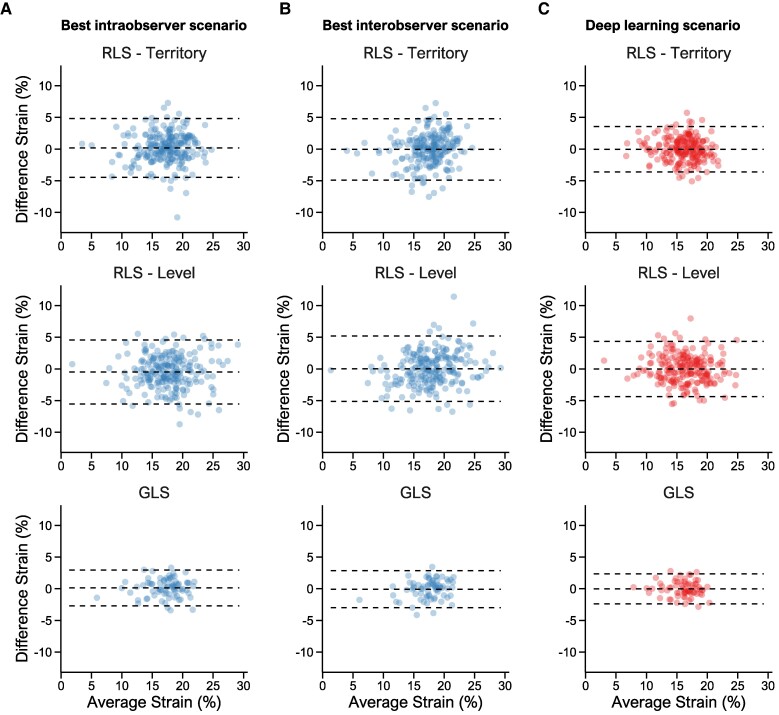
The agreement of strains in repeated recordings by regions, methods, and selected observer scenarios. The Bland–Altman plots show the average and difference of strains for repeated recordings. The dashed lines show bias and LOA. Column *(A)* shows values for the best intraobserver scenario, Column *(B)* shows values for the best interobserver scenario, and Column *(C)* shows values for the DL method. Abbreviations: as in *Figure 1*.

The differences in test–retest values for both RLS_Territory_ and RLS_Level_ demonstrated a significantly lower variance for the DL method compared with all observer scenarios (all *P* < 0.001). The DL method also had lower absolute differences for all strains compared with the observer scenarios, with the median absolute differences of 1.2 vs. 1.8% (RLS_Territory_), 1.4 vs. 1.7% (RLS_Level_), and 0.7 vs. 1.4% (GLS) for the DL method and interobserver scenarios, respectively (*Figure 2*). The improved reproducibility by the DL method for RLS_Territory_, RLS_Level_, and GLS was applicable to the whole range of strain values (*Figure 3*). For RLS_Territory_ and GLS, the MDC was slightly higher in the control group than in the patient group, whereas it was similar between groups for RLS_Level_. However, the MDC was consistently lower for DL compared with human observers (see Supplementary data online, *Table S2*).

### Strain measurements by methods

Both the DL method and the semi-automatic method used by the three observers showed small, but significant differences in measured strain values within each of the two consecutive recordings for RLS_Territory_, RLS_Level_, and GLS (all *P* < 0.001). Pairwise comparisons confirmed different strain values for all combinations of the DL method and the observers, except for Observers 1 and 2 (*P* = 0.99 and *P* = 0.98, respectively, *Table 3* and Supplementary data online, *Figures S2*–*S8*). The DL method used 1.0 ± 0.3 s to measure LV strain for one cardiac cycle per subject and measured 3.1 ± 0.7 cardiac cycles per subject.

**Table 3:** Strain measurements according to methods, observers, and recordings

	Semi-automatic method			Deep learning method
	Observer 1	Observer 2	Observer 3	
Recording 1				
LAD territory	17.6 ± 3.4	17.7 ± 2.8	18.7 ± 3.4	14.9 ± 2.7
CX territory	17.0 ± 3.5	17.7 ± 3.1	17.7 ± 3.8	16.1 ± 3.0
RCA territory	16.8 ± 3.6	17.2 ± 3.5	18.5 ± 3.8	17.0 ± 2.9
Basal level	14.2 ± 3.3	15.2 ± 3.0	15.0 ± 3.6	14.1 ± 3.5
Mid-level	18.3 ± 3.3	17.9 ± 2.7	18.6 ± 3.4	15.9 ± 3.0
Apical level	20.6 ± 4.8	20.5 ± 4.3	22.8 ± 5.0	18.7 ± 3.4
GLS	17.2 ± 3.0	17.5 ± 2.6	18.3 ± 3.2	15.9 ± 2.5
Recording 2				
LAD territory	17.8 ± 3.2	16.9 ± 3.1	18.6 ± 3.3	15.2 ± 2.8
CX territory	17.2 ± 3.7	17.6 ± 3.5	18.0 ± 3.9	16.1 ± 2.8
RCA territory	16.8 ± 3.8	16.7 ± 3.5	18.6 ± 4.0	17.0 ± 2.9
Basal level	14.6 ± 3.4	15.0 ± 3.1	15.6 ± 3.7	14.3 ± 3.1
Mid-level	17.7 ± 3.4	17.2 ± 2.9	18.6 ± 3.2	15.8 ± 2.9
Apical level	20.7 ± 4.7	19.9 ± 4.4	22.5 ± 5.0	18.9 ± 3.1
GLS	17.2 ± 3.0	17.1 ± 2.9	18.4 ± 3.2	16.0 ± 2.5

The mean ± SD of measured peak systolic strain (%, absolute values) for the two consecutive recordings.

GLS, global longitudinal strain; LAD, left anterior descending coronary artery; CX, circumflex coronary artery; RCA, right coronary artery.

### Feasibility

The feasibility of strain measurements was excellent with 93% of all segments analysed by the DL method and 93–98% by the three observers using the semi-automatic method.

## Discussion

This study evaluated the reproducibility of a novel DL-based method for automated RLS measurements in a test–retest scenario using repeated echocardiographic recordings. The main findings of this study were as follows: (i) the DL method provided superior test–retest reproducibility of RLS compared with three experienced observers using a semi-automatic reference method; (ii) the DL method measured RLS and GLS fully automated, fast, and with high feasibility; (iii) the reproducibility of RLS measured by the DL method matched the interobserver reproducibility of GLS measured using commercial semi-automatic software, which is generally accepted for clinical use. This suggests that the novel DL method has potential to advance patient care through a more reliable and time-efficient assessment of regional myocardial function.

### Test–retest reproducibility of RLS of this study compared with that of previous studies

Previous studies on reproducibility of RLS focused on evaluating the variability within the same echocardiographic recording,^21–23^ whereas this study assessed the variability in consecutive echocardiographic recordings combined with variations in the strain measurement procedure. Repeated measurements in the same recording do not necessarily reflect the clinically relevant variability in echocardiography. This is also true for other echocardiographic measurements, and a recent study showed improved test–retest reproducibility of LVEF by a DL method, with an MDC of 16.2, 12.8, and 12.0%, respectively, for the interobserver, intraobserver, and DL scenarios.^24^ Few studies have assessed the total variability induced by variations in consecutive echocardiographic recordings combined with variations in the strain measurement procedure.^5,6,25,26^ Chamberlain *et al.*^5^ analysed RLS measurements in 40 subjects with consecutive echocardiographic recordings using ultrasound machines from different vendors, where strain was measured by the same observer in both recordings using vendor-independent software. Similarly, Shiino *et al.*^6^ measured RLS in a test–retest setting using vendor-dependent software and echocardiograms from 55 subjects. Both these studies presented LOAs for RLS_Territory_ and RLS_Level_ which were on average similar to, or wider than, the observers’ semi-automatic measurements in our study. This highlights the robustness of the reference measurements in the present study (*Table 2*). The relative improvement in reproducibility may be even higher if the RLS measurements of the DL method were compared with semi-automatic measurements acquired in clinical, less standardized settings.

### DL-based methods for measuring myocardial strain

Several DL-based applications exist for measuring GLS in echocardiography, with recent publications assessing diagnostic performance, agreement with manual measurements, and reproducibility of both global and regional strains.^27–29^ However, few publications have evaluated their test–retest performance, which is critical for the clinical application of new methods. To the best of our knowledge, this is the first study to assess the test–retest reproducibility of RLS using a fully automated DL method.

In comparison with the previous optical flow–based motion estimator DL method for measuring GLS published by our group, the refined DL method incorporates a novel learning-based point tracking algorithm. This evolution not only allowed for reliable measurements of RLS, but also improved the reproducibility of GLS by 36% compared with our previous method (MDC 2.3 vs. 3.6%), while simultaneously decreasing GLS measurement time by 87% (1.0 vs. 7.9 s).^13^ The superior reproducibility of the DL method compared with semi-automatic methods might partly be explained by removing the user-dependent ROI initialization. Moreover, DL-based methods outperform traditional speckle tracking by generalizing well across diverse scenes, learning rich feature representations directly from data, and optimizing for challenging scenarios with intricate motion, noisy images, speckle decorrelation, and target drift.

### Future perspectives

Quantification of LV function by GLS has previously demonstrated both diagnostic and prognostic value in various cardiac diseases.^21,22,30^ The clinical value of GLS might partly be explained by its high reproducibility, regardless of echocardiographic training.^12^ As non-uniform myocardial involvement is common in many cardiac diseases, a highly reproducible assessment of regional deformation may improve patient care. One important example is acute and chronic coronary artery disease, where RLS_Territory_ correlates with infarct size^31^ and can identify persistent occlusion of the infarct-related artery in patients with non-ST segment elevation acute coronary syndrome.^21,23,32^ Furthermore, rapid and fully automated analyses of RLS_Territory_ might have potential to improve the clinical utility of stress echocardiography. Conversely, RLS_Level_ provides diagnostic value in patients with cardiomyopathies, cardiac amyloidosis, takotsubo syndrome, and more.^33–36^ The diagnostic potential and prognostic potential of DL-based RLS measurements should be explored in future clinical studies.

Successful clinical implementation and user trust in DL-based applications rely on transparency and explainability. This can be achieved through user oversight and quality control by managing ROI initialization and tracking, as well as the ability to adjust or exclude measurements if needed. Using the presented DL method, the results for all steps in the method can easily be overseen by the human user before clinical decision-making. In the present study, however, the DL method operated fully automated without any observer input.

### Strengths and limitations

Major strengths of this study include the dual-centre test–retest design and utilizing two consecutive echocardiograms per subject, which increases generalizability and minimizes physiological variability. The novel DL method is vendor independent and can, in principle, be applied to echocardiograms from any vendor. However, in the present study we only used data from one vendor. The included echocardiograms were acquired using two generations of ultrasound scanners (GE Vivid E95 and Vivid 7), which strengthens the generalizability of our results since previous generations are commonly used worldwide. The mean strains measured by the DL method were lower than those measured by the semi-automatic method. However, this bias was of comparable magnitude to that previously demonstrated with strain software from different vendors.^4^ The categorization of myocardial segments into predefined coronary artery territories is only theoretical and may not be valid at the level of individual patients.^37^ RLS_Territory_ does, however, provide a more detailed differentiation of regional myocardial function than GLS. Although even more detailed data on a segmental level might be of clinical interest, further technical work and thorough validation are still required. This will be important to evaluate in future studies.

## Conclusion

The novel DL method based on point tracking technology provided measurements of RLS for regions corresponding to the perfusion territories of the coronary arteries, and the basal-to-apical levels of the LV, with superior test–retest reproducibility compared with the semi-automatic measurements conducted by experienced observers. With its fully automated approach, the DL-based tool might have significant implications for clinical practice through a more detailed, more efficient, and less user-dependent assessment of myocardial function.

## Supplementary Material

qyae092_Supplementary_Data

## Data Availability

The data underlying this article will be shared on reasonable request to the corresponding author.

## Social media

#DeepLearning improves the reproducibility of regional strain in #echofirst. With precision approaching GLS measured by semi-automatic speckle tracking–based methods, #DeepLearning enables a more efficient and consistent evaluation of regional myocardial function. @CIUS_NTNU, @ProCardio1, @StOlavshospital.

## Lead author biography

John Nyberg completed medical school at Karolinska Institutet in Stockholm, Sweden, followed by an internship at Herlev Hospital and Bispebjerg Hospital in Copenhagen, Denmark. Thereafter, he worked as a cardiology resident at Zealand University Hospital, Roskilde and Køge, Denmark, before enrolling as a PhD candidate at the Norwegian University of Science and Technology in 2021. His research focuses on the development and clinical validation of new methods for quantifying myocardial function using echocardiographic deformation imaging and deep learning–based methods.
