# Supplementary material for: Deep learning improves test–retest reproducibility of regional strain in echocardiography
Source: Eur Heart J Imaging Methods Pract. 2024 Oct 23;2(4):qyae092. doi: 10.1093/ehjimp/qyae092 (PMC11498295; doi:10.1093/ehjimp/qyae092)
Supplement: qyae092_Supplementary_Data [file qyae092_supplementary_data.docx]

Supplementary material to:

*Deep Learning Improves Test-Retest Reproducibility of Regional Strain in Echocardiography*

[Supplementary Table 1. Test-retest reliability metrics for strain from all intra-operator and inter-operator scenarios 3](#_Toc174704260)

[Supplementary Table 2. Minimal detectable change per dataset, region and method 5](#_Toc174704261)

[Supplementary Figure 1. Myocardial segments and the calculation of RLS – Territory. 6](#_Toc174704262)

[Supplementary Figure 2. GLS values according to methods, operators and recordings. 7](#_Toc174704263)

[Supplementary Figure 3. RLS for LAD territory according to methods, operators and recordings. 8](#_Toc174704264)

[Supplementary Figure 4. RLS for CX territory according to methods, operators and recordings. 9](#_Toc174704265)

[Supplementary Figure 5. RLS for RCA territory according to methods, operators and recordings. 10](#_Toc174704266)

[Supplementary Figure 6. RLS for basal level according to methods, operators and recordings. 11](#_Toc174704267)

[Supplementary Figure 7. RLS for mid level according to methods, operators and recordings. 12](#_Toc174704268)

[Supplementary Figure 8. RLS for apical level according to methods, operators and recordings. 13](#_Toc174704269)

[Supplementary Methods 14](#_Toc174704270)

### Supplementary Table 1. Test-retest reliability metrics for strain from all intra-operator and inter-operator scenarios

|  | **MDC** | **ICC** | **Bias** | **LOA**  **(lower)** | **LOA**  **(higher)** | **CoV** |
| --- | --- | --- | --- | --- | --- | --- |
| **RLS - Territory** | | | | | | |
| Deep learning | 3.6 | 0.89 | 0.0 | -3.6 | 3.6 | 5 |
| Interobserver | 4.8 | 0.85 | -0.1 | -4.9 | 4.8 | 6 |
| Interobserver | 4.9 | 0.82 | -0.2 | -5.1 | 4.8 | 6 |
| Interobserver | 5.7 | 0.76 | 1.4 | -3.6 | 6.4 | 7 |
| Interobserver | 5.7 | 0.78 | -1.1 | -6.4 | 4.2 | 7 |
| Interobserver | 5.8 | 0.74 | 1.1 | -4.3 | 6.4 | 7 |
| Interobserver | 6.3 | 0.71 | -1.4 | -7.0 | 4.2 | 7 |
| Intraobserver | 4.6 | 0.89 | 0.2 | -4.5 | 4.8 | 6 |
| Intraobserver | 4.8 | 0.81 | -0.4 | -5.2 | 4.3 | 6 |
| Intraobserver | 5.4 | 0.83 | 0.1 | -5.3 | 5.5 | 6 |
| **RLS - Level** | | | | | | |
| Deep learning | 4.3 | 0.89 | 0.0 | -4.4 | 4.4 | 6 |
| Interobserver | 5.2 | 0.85 | 0.0 | -5.1 | 5.2 | 6 |
| Interobserver | 5.9 | 0.79 | -0.4 | -6.3 | 5.5 | 7 |
| Interobserver | 6.2 | 0.72 | 1.2 | -4.5 | 7.0 | 7 |
| Interobserver | 6.4 | 0.74 | 1.4 | -4.4 | 7.1 | 7 |
| Interobserver | 7.1 | 0.73 | -1.2 | -7.9 | 5.4 | 7 |
| Interobserver | 7.6 | 0.66 | -1.6 | -8.6 | 5.3 | 8 |
| Intraobserver | 5.1 | 0.80 | -0.5 | -5.5 | 4.6 | 6 |
| Intraobserver | 5.3 | 0.86 | 0.0 | -5.3 | 5.4 | 6 |
| Intraobserver | 5.7 | 0.83 | 0.1 | -5.7 | 5.8 | 6 |
| **GLS** |  |  |  |  |  |  |
| Deep learning | 2.3 | 0.89 | 0.0 | -2.4 | 2.4 | 3 |
| Interobserver | 2.9 | 0.86 | -0.1 | -3.0 | 2.9 | 4 |
| Interobserver | 3.5 | 0.81 | -0.2 | -3.7 | 3.3 | 4 |
| Interobserver | 4.1 | 0.74 | 1.1 | -2.4 | 4.6 | 5 |
| Interobserver | 4.2 | 0.77 | -1.1 | -4.7 | 2.4 | 5 |
| Interobserver | 4.3 | 0.75 | 1.4 | -1.9 | 4.7 | 5 |
| Interobserver | 4.6 | 0.71 | -1.4 | -5.1 | 2.3 | 6 |
| Intraobserver | 2.8 | 0.89 | 0.1 | -2.7 | 3.0 | 4 |
| Intraobserver | 3.4 | 0.81 | -0.5 | -3.7 | 2.8 | 4 |
| Intraobserver | 3.6 | 0.83 | 0.1 | -3.5 | 3.8 | 4 |

All metrics are reported as strain percentages (%) except ICC which is unitless and CoV which is relative percentage. Abbreviations: MDC, minimal detectable change; ICC, intraclass correlation coefficient; LOA limits of agreement; CoV, coefficient of variability.

### Supplementary Table 2. Minimal detectable change per dataset, region and method

| **Dataset** | **Region** | **Deep learning** | **Interobserver** | **Intraobserver** |
| --- | --- | --- | --- | --- |
| HUNT4Echo | RLS - Territory | 3.8 | 5.7 (5.1 to 6.2) | 5.3 (5.0 to 5.7) |
| HUNT4Echo | RLS - Level | 4.3 | 6.2 (5.5 to 7.1) | 5.7 (5.4 to 6.1) |
| HUNT4Echo | GLS | 2.8 | 3.8 (2.8 to 4.2) | 3.4 (3.2 to 3.5) |
| Hospital of Southern Norway | RLS - Territory | 3.4 | 5.4 (4.5 to 6.3) | 4.6 (4.2 to 5.1) |
| Hospital of Southern Norway | RLS - Level | 4.3 | 6.6 (4.8 to 8.8) | 5.1 (4.6 to 6.0) |
| Hospital of Southern Norway | GLS | 1.9 | 4.1 (2.9 to 5.2) | 3.2 (2.5 to 3.8) |

Mean (range) of minimal detectable change are given for interobserver and intraobserver scenarios. Minimal detectable change is reported as strain percentage (%).

### Supplementary Figure 1. Myocardial segments and the calculation of RLS – Territory.


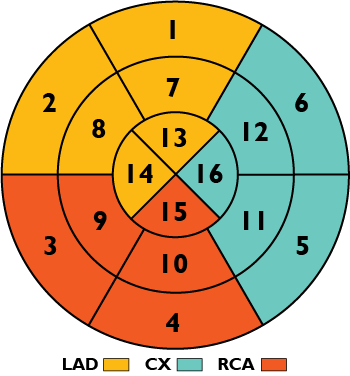


Supplementary Figure 1 legend: Schematic drawing of segments included in the three coronary artery perfusion territories. The six basal myocardial segments are: 1, anterior; 6, lateral; 5 posterior; 4 inferior; 3 septal, 2 anteroseptal. The six mid myocardial segments are: 7, anterior; 12, lateral; 11 posterior; 10 inferior; 9 septal, 8 anteroseptal. The four apical myocardial segments are: 13, anterior, 16 average of lateral and posterior; 15 inferior; 14 average of septal and anteroseptal. Abbreviations: RLS. regional longitudinal strain, LAD, left anterior descending coronary artery; CX, circumflex coronary artery; RCA, right coronary artery

### Supplementary Figure 2. GLS values according to methods, operators and recordings.


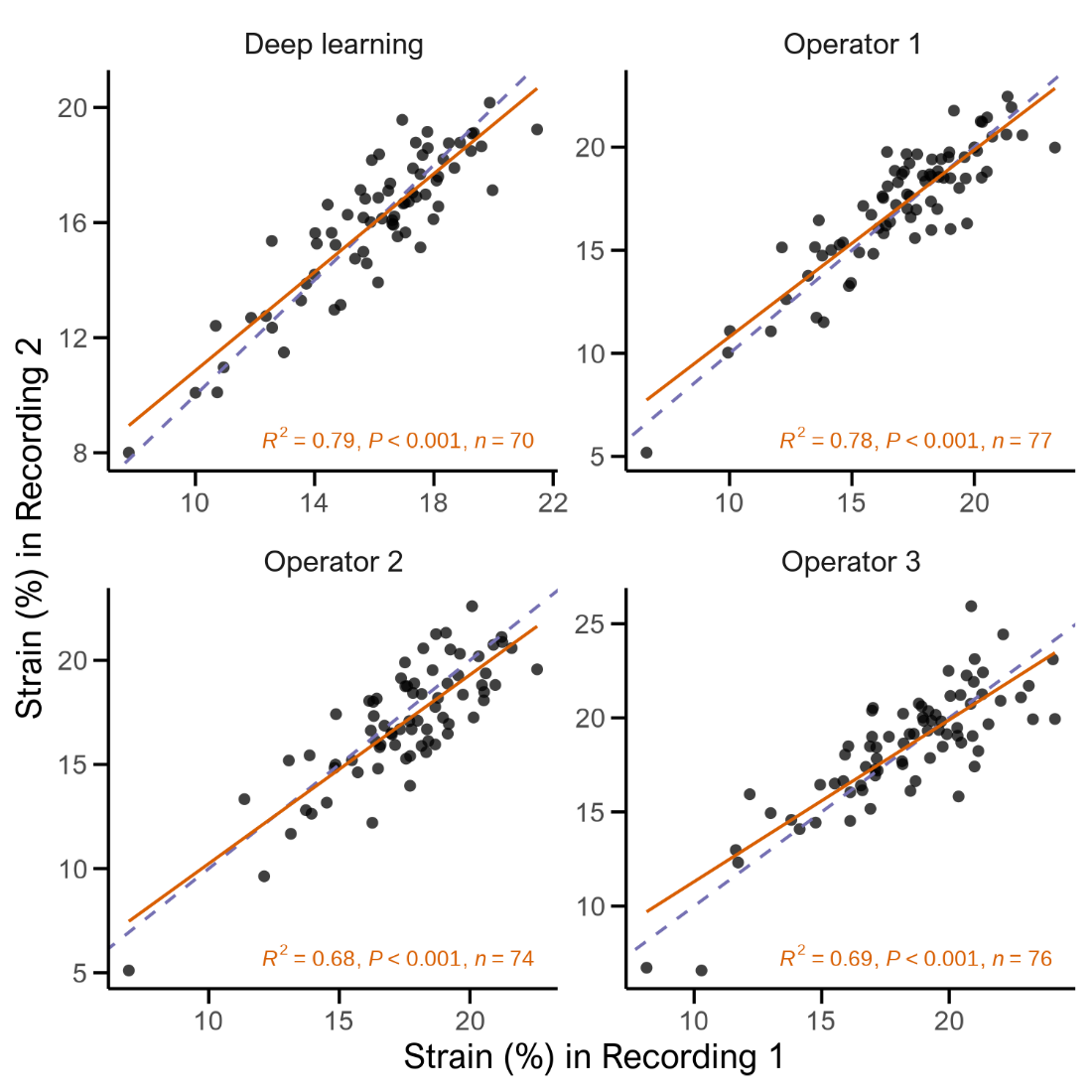


Supplementary Figure 2 Legend: Scatterplots of GLS measurements for Recording 1 (x-axis) vs Recording 2 (y-axis) by the DL method, and the three operators. Red solid line shows regression line and blue dashed line is line of equality. Abbreviations: R^2^, coefficient of determination, otherwise as in Supplementary Table 1.

### Supplementary Figure 3. RLS for LAD territory according to methods, operators and recordings.


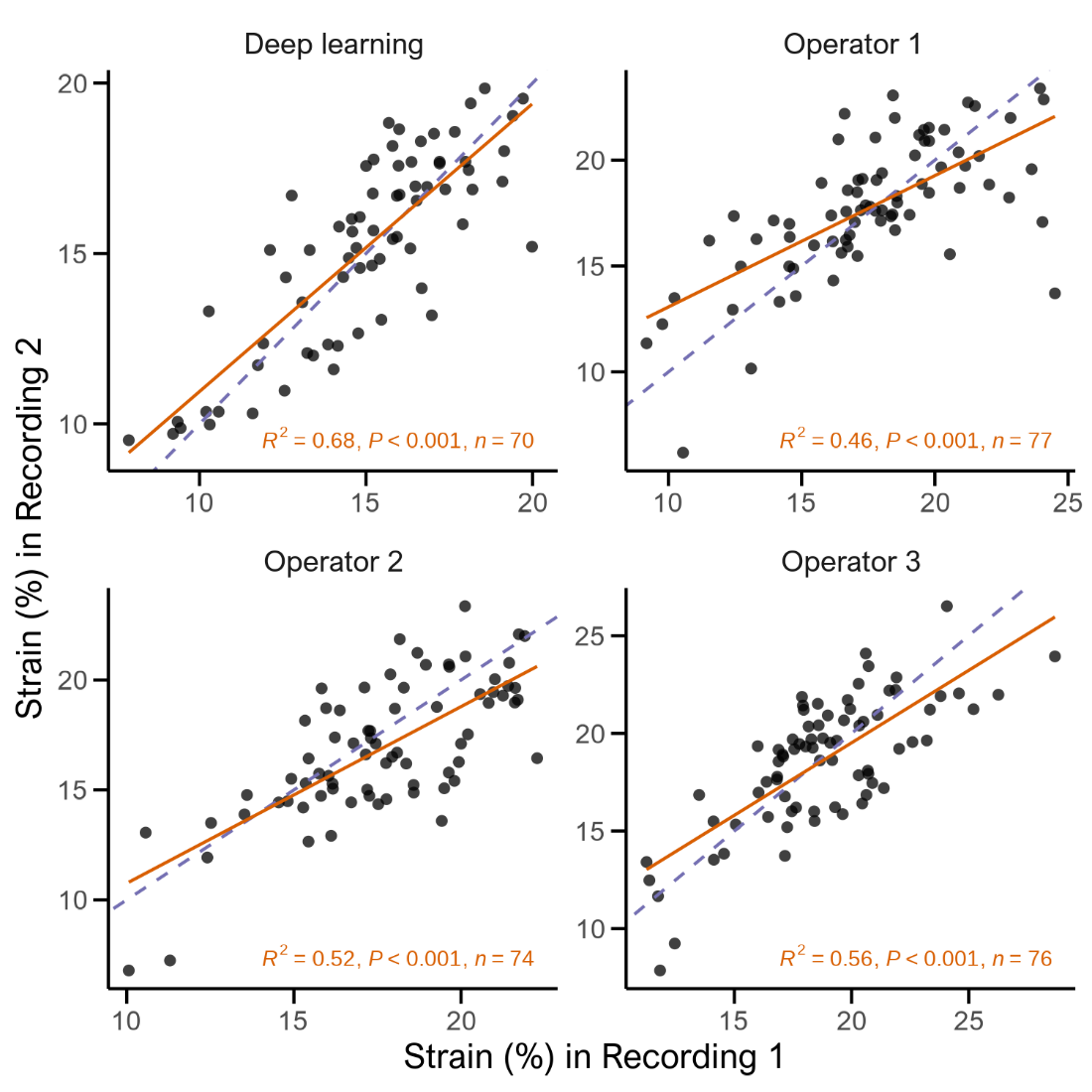


Supplementary Figure 3 Legend: Scatterplots of RLS measurements for the LAD territory for Recording 1 (x-axis) vs Recording 2 (y-axis) by the DL method, and the three operators. Red solid line shows regression line and blue dashed line is line of equality. Abbreviations: R^2^, coefficient of determination, otherwise as in Supplementary Table 1.

### Supplementary Figure 4. RLS for CX territory according to methods, operators and recordings.


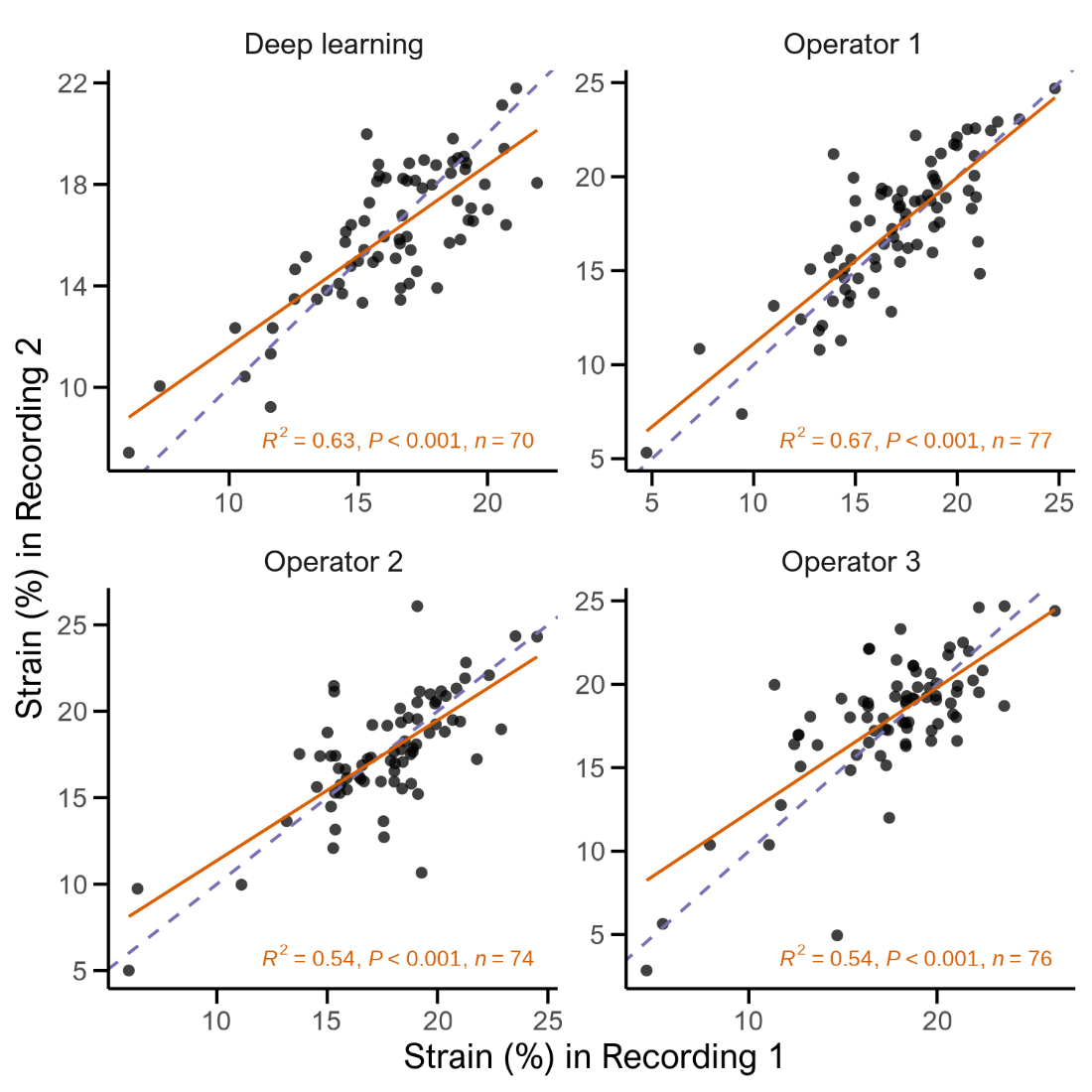


Supplementary Figure 4 Legend: Scatterplots of RLS measurements for the CX territory for Recording 1 (x-axis) vs Recording 2 (y-axis) by the DL method, and the three operators. Red solid line shows regression line and blue dashed line is line of equality. Abbreviations: R^2^, coefficient of determination, otherwise as in Supplementary Table 1.

### Supplementary Figure 5. RLS for RCA territory according to methods, operators and recordings.


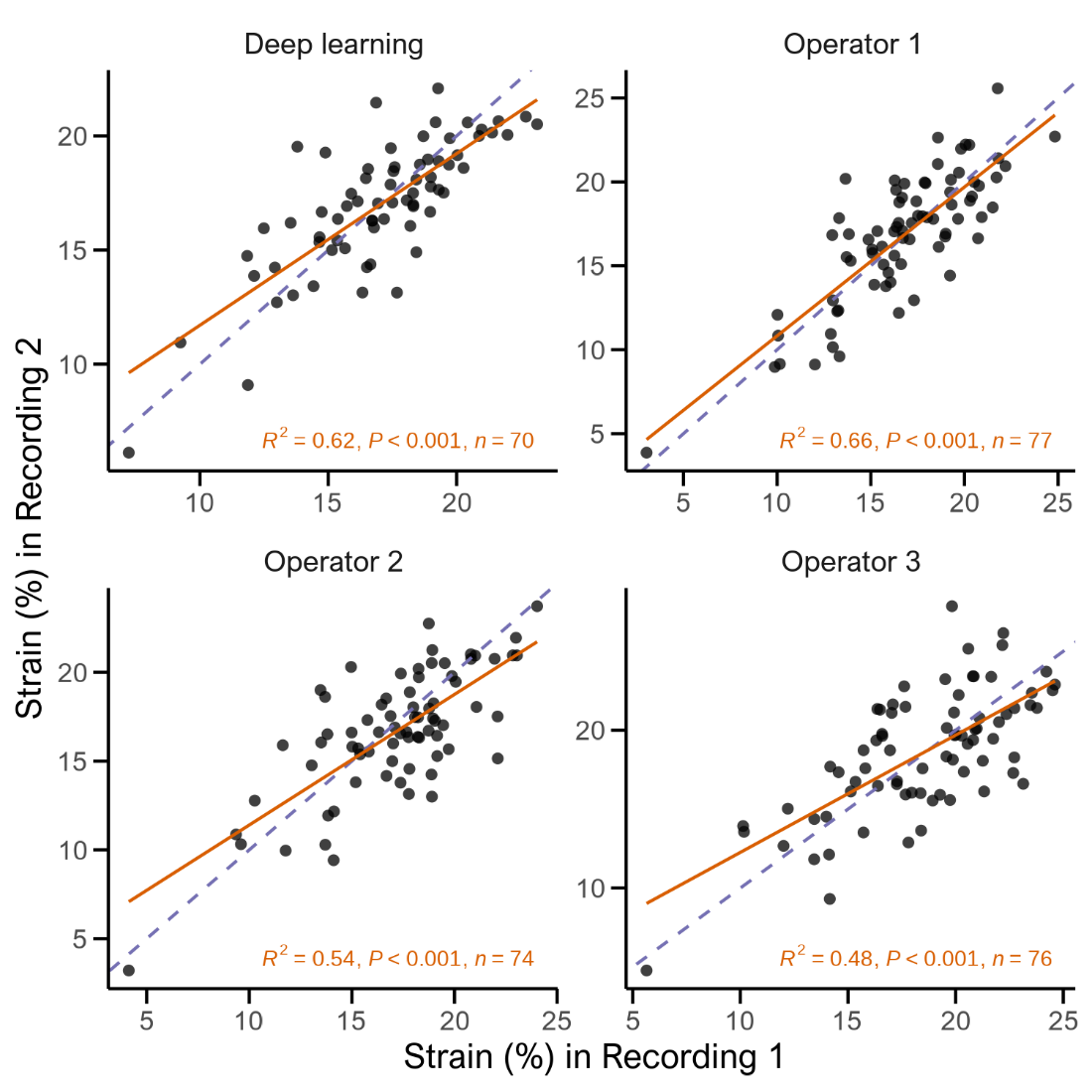


Supplementary Figure 5 Legend: Scatterplots of RLS measurements for the RCA territory for Recording 1 (x-axis) vs Recording 2 (y-axis) by the DL method, and the three operators. Red solid line shows regression line and blue dashed line is line of equality. Abbreviations: R^2^, coefficient of determination, otherwise as in Supplementary Table 1.

### Supplementary Figure 6. RLS for basal level according to methods, operators and recordings.


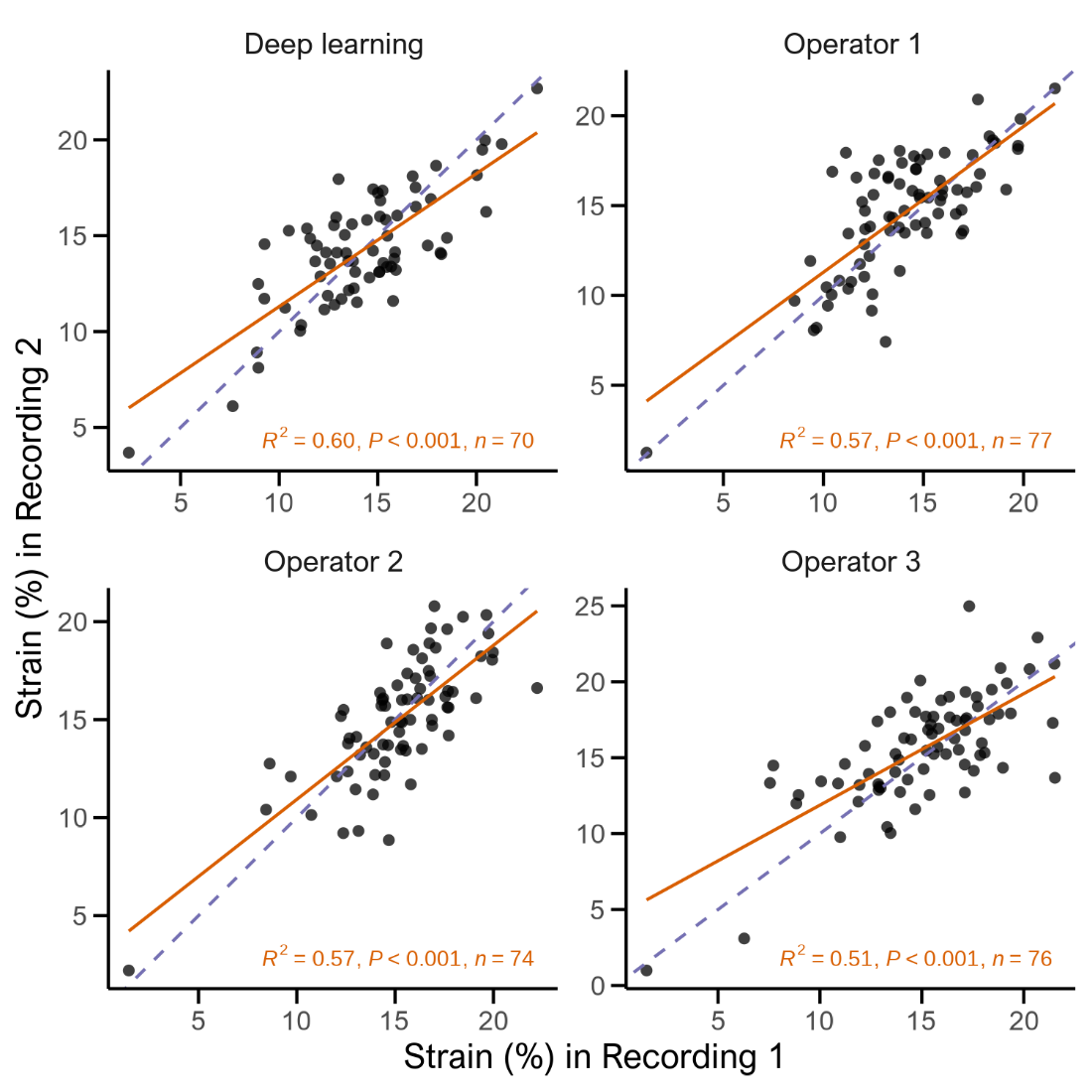


Supplementary Figure 6 Legend: Scatterplots of RLS measurements for the basal level for Recording 1 (x-axis) vs Recording 2 (y-axis) by the DL method, and the three operators. Red solid line shows regression line and blue dashed line is line of equality. Abbreviations: R^2^, coefficient of determination, otherwise as in Supplementary Table 1.

### Supplementary Figure 7. RLS for mid level according to methods, operators and recordings.


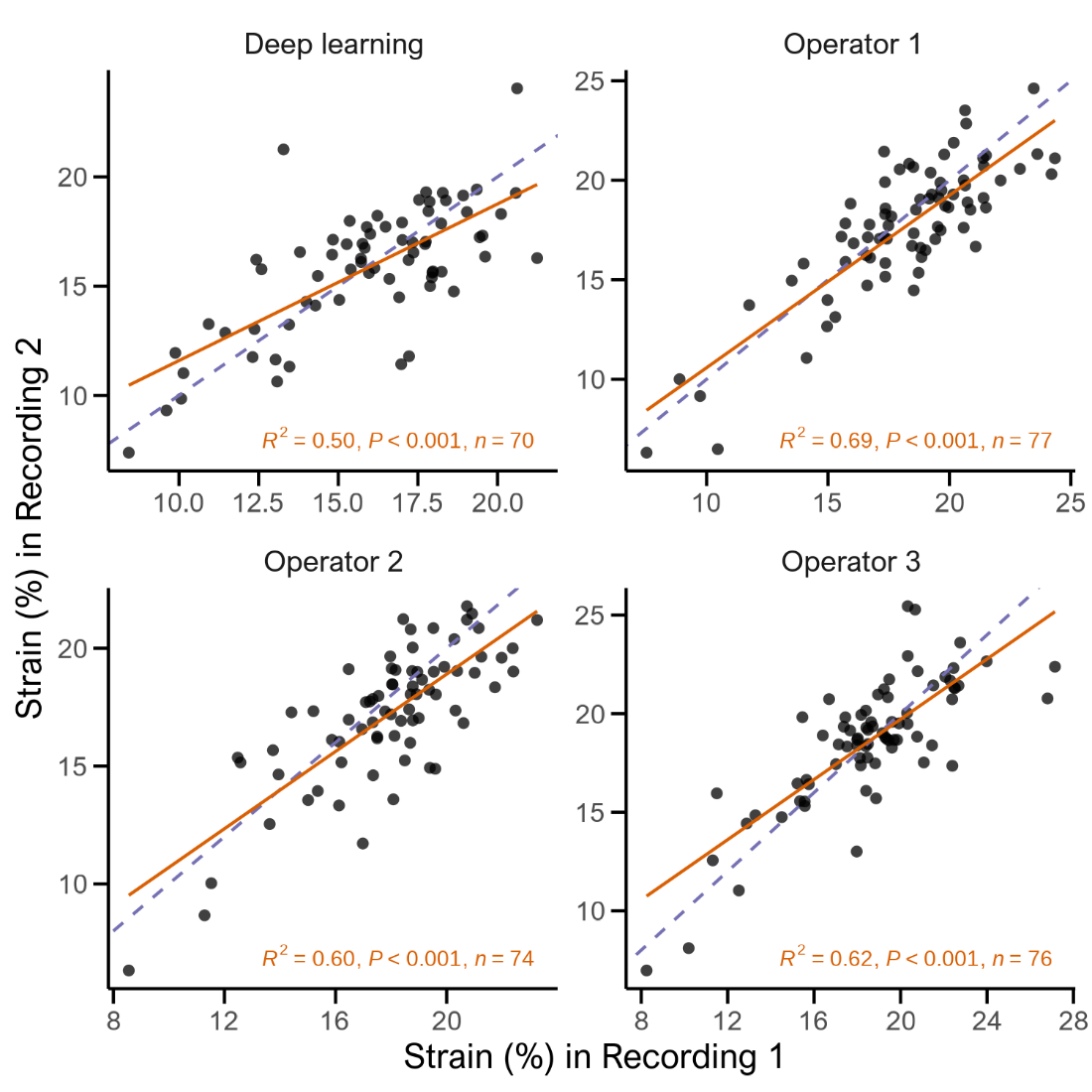


Supplementary Figure 7 Legend: Scatterplots of RLS measurements for the mid level for Recording 1 (x-axis) vs Recording 2 (y-axis) by the DL method, and the three operators. Red solid line shows regression line and blue dashed line is line of equality. Abbreviations: R^2^, coefficient of determination, otherwise as in Supplementary Table 1.

### Supplementary Figure 8. RLS for apical level according to methods, operators and recordings.


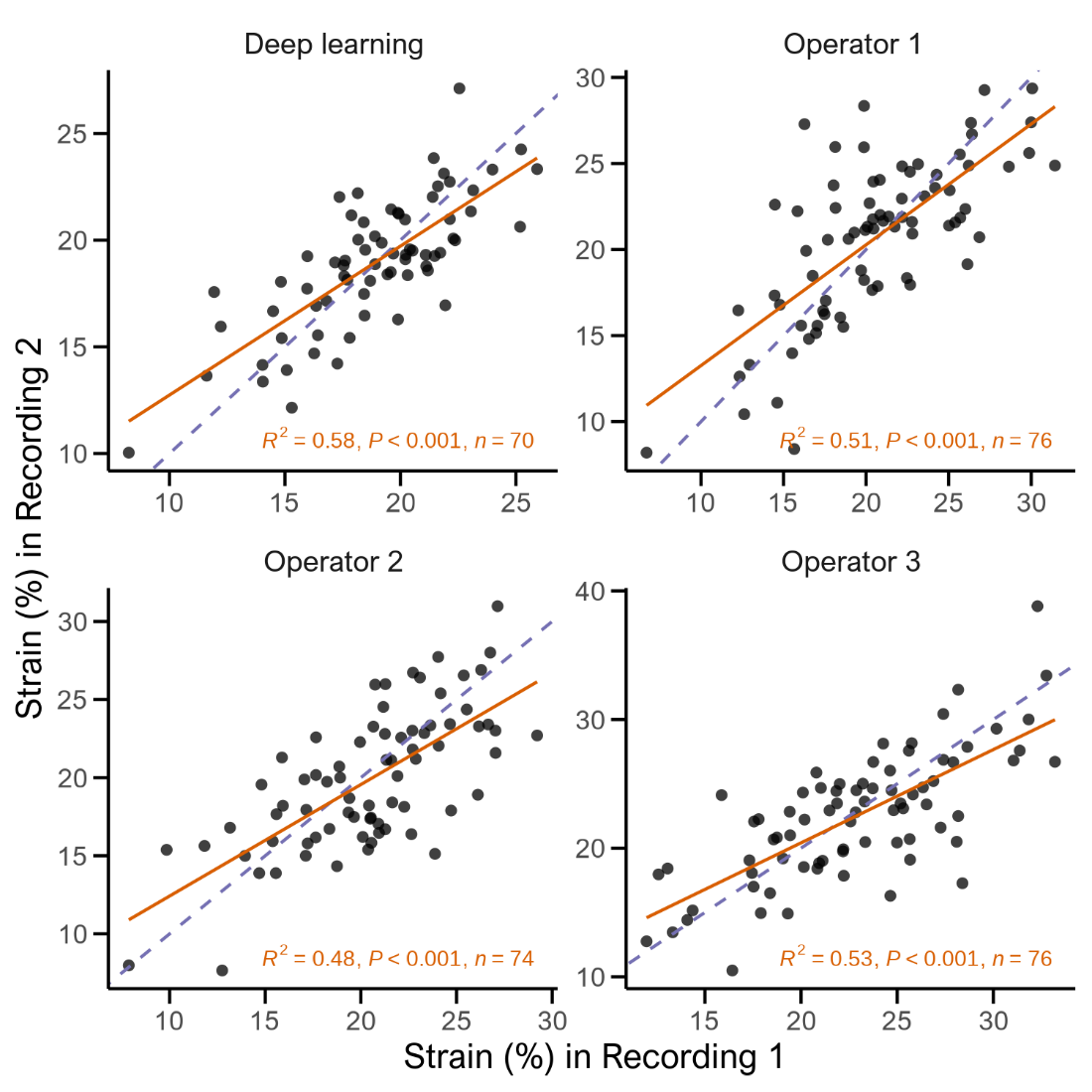


Supplementary Figure 8 Legend: Scatterplots of RLS measurements for the apical level for Recording 1 (x-axis) vs Recording 2 (y-axis) by the DL method, and the three operators. Red solid line shows regression line and blue dashed line is line of equality. Abbreviations: R^2^, coefficient of determination, otherwise as in Supplementary Table 1.

### Supplementary Methods

The Nord-Trøndelag Health Study – Echocardiographic sub-study (HUNT4Echo) is a population-based cohort study ongoing since 1984. HUNT4 is the 4th wave of the study and included the echocardiographic substudy (HUNT4Echo), evaluating a subgroup of 2,462 study participants between 2017 and 2018. Out of these 2,462 study participants, 40 underwent sequential echocardiographic examinations by either a sonographer or a cardiologist. A Vivid E95 Scanner was used for all recordings (GE Healthcare, Horten, Norway).

The clinical echocardiographic study conducted at Hospital of Southern Norway, Arendal during 2013 is constituted of a study population with surviving patients with previous suspected acute coronary syndrome. Patients from this cohort underwent sequential echocardiographic examinations conducted by either a cardiology resident or and cardiologist. The two operators were blinded for each other’s recordings. A Vivid 7 Scanner was used for all recordings (GE Healthcare, Horten, Norway). A total of 47 patients were included and complete echocardiograms from both operators were available for 40 of them.
